# Long-Term Prediction of the Arctic Ionospheric TEC Based on Time-Varying Periodograms

**DOI:** 10.1371/journal.pone.0111497

**Published:** 2014-11-04

**Authors:** Jingbin Liu, Ruizhi Chen, Zemin Wang, Jiachun An, Juha Hyyppä

**Affiliations:** 1 Department of remote sensing and photogrammetry, Finnish Geodetic Institute, Masala, Finland; 2 Conrad Blucher Institute for surveying & science, Texas A & M University Corpus Christi, Corpus Christi, United States of America; 3 Chinese Antarctic center of surveying and mapping, Wuhan University, Wuhan, China; University of Oxford, United Kingdom

## Abstract

Knowledge of the polar ionospheric total electron content (TEC) and its future variations is of scientific and engineering relevance. In this study, a new method is developed to predict Arctic mean TEC on the scale of a solar cycle using previous data covering 14 years. The Arctic TEC is derived from global positioning system measurements using the spherical cap harmonic analysis mapping method. The study indicates that the variability of the Arctic TEC results in highly time-varying periodograms, which are utilized for prediction in the proposed method. The TEC time series is divided into two components of periodic oscillations and the average TEC. The newly developed method of TEC prediction is based on an extrapolation method that requires no input of physical observations of the time interval of prediction, and it is performed in both temporally backward and forward directions by summing the extrapolation of the two components. The backward prediction indicates that the Arctic TEC variability includes a 9 years period for the study duration, in addition to the well-established periods. The long-term prediction has an uncertainty of 4.8–5.6 TECU for different period sets.

## Introduction

As a result of climate change, the industrial and political importance of the Arctic area is growing significantly, and human activities are currently increasing in the Arctic region, including marine, terrestrial and space domains. In the Earth's ionosphere circulation, the polar ionosphere is located at the frontline of those areas responding to variations in the solar-terrestrial physical system because the polar ionosphere is directly connected to the interplanetary space and the Sun. The Total Electron Content (TEC) is an important parameter of the Earth's ionosphere. More detailed knowledge, modeling and predictions of Arctic TEC variability are of fundamental relevance in both engineering and science. Monitoring and predicting the Earth's ionosphere are among major tasks of the fields of solar-terrestrial physics and space weather [1]–[4]. In engineering fields, long-term predictions of the ionosphere over the scale of a decade can aid in evaluations of ionospheric effects on numerous radio navigation and communication systems as the ionosphere, which responses to solar activities, influences the technical systems in various ways: posing hazards to satellites, disrupting power-grids, causing blackouts in radio and telecommunication systems, even affecting the astronauts in space; predictions of ionosphere and other space weather are also required for guaranteeing effective operation, planning, and risk management of satellite and space exploration missions on time scales ranging from days to weeks to a solar cycle [5]–[6] because all of satellites and spacecraft are sensitive at some level to ionosphere and solar cycle effects [3], [7]–[8]. These kinds of space weather predictions will continue in research and operational settings in future, and the need for these predictions has moved from the science community to a global space weather user support system [8]. As an increasing number of countries are planning and implementing their satellite and space exploration missions, there is the need for any country with assets in space to monitor and predict space weather, including ionosphere condition and solar cycle, to protect their satellites and technology.

With the efforts of the International GNSS (Global Navigation Satellite Systems) Service (IGS) and geophysical research communities over the past two decades, the Global Positioning System (GPS) has become an endorsed ionosphere observation tool due to its ability to continuously observe the Earth's ionosphere over large spatial scales [9]–[13]. The IGS Ionosphere Working Group has constructed databases of GPS observables and TEC products derived from a continuously operating global network of ground-based GPS receivers [11], [14]. Based on these long-term GPS TEC products, ionosphere climatology has recently been investigated on regional and global scales, as in the works [15]–[17]. In these studies, the time evolution of periodograms of regional and global TEC was reported, and empirical models of the ionospheric TEC were correspondingly constructed using input solar and geophysical indices, including Extreme Ultraviolet (EUV) irradiance, the 10.7 cm solar radio flux (*F*10.7) index and the geomagnetic activity index. Previously an empirical model can reconstruct past ionospheric TEC data using past solar and geophysical indices; however, it cannot predict future TEC values without physical data input [16].

The state of the ionosphere can be predicted by either extrapolation methods or physical models [18]–[20]. Past studies have developed various empirical models to predict one or more physical parameters of the ionosphere on short time scales from days to weeks [21]. For example, the Advanced Stand Alone Prediction System allows for the prediction of radio communication conditions in the high-frequency and very-high-frequency radio spectrum, while the Ionospheric Communications Enhanced Profile Analysis and Circuit model predicts the maximum usable frequency parameter using the electron density profile. The widely acknowledged International Reference Ionosphere model utilizes predicted physical indices to provide expected ionospheric parameters and electron density profiles and can further predict the ionospheric TEC for given locations and dates in a short time scale [22]. An extrapolation method was developed to directly predict the global mean TEC for the next 2–7 years based on a stationary spectral analysis of GPS-derived TEC data for the past four years, which represented all of the data available at that time [14].

Based on the recent findings, this work develops a new extrapolation method of predicting the evolution of Arctic TEC parameters by utilizing time-varying periodograms of the variability of the Arctic TEC. Compared to the previous extrapolation method, this study uses data of the past 13.6 years, which allows us to analyze the periodograms of the TEC variability on the scale of a solar cycle [14]. The newly developed method in this study utilizes the time-varying periodograms of the Arctic TEC to perform long-term prediction over a solar cycle. The TEC time series is first divided into a component of periodic oscillations and a component of the average TEC, in addition to noise. This study investigates the variability of both components separately and forecasts the two components over the scale of a solar cycle. The prediction is conducted in both temporally backward and forward directions. The results of the backward prediction are compared with existing data to verify the performance of the prediction.

In this paper, Section 2 introduces the method of Spherical Cap Harmonic Analysis (SCHA) used to map the Arctic TEC and to estimate the Arctic mean TEC. The periodograms of the Arctic TEC variability are then investigated. Finally, the Arctic TEC values are predicted.

## Methods and Materials of SCHA Mapping of the Regional Ionospheric TEC

The ionospheric TEC along GPS signal paths can be estimated using dual-frequency GPS observables, satellite orbit products, and the hardware delay parameters of receivers and satellites. The method of estimating the vertical TEC from GPS measurements has been presented in a number of works [2], [23]–[25]. The previous work has developed a technique of carrier-phase smoothing to improve the accuracy of pseudorange-derived TEC estimates [26]. The estimated TEC data are used with the SCHA model to map the regional ionospheric TEC. In this work, a spherical cap is a regional part of a sphere, and it is defined by the geographical coordinate of a spherical cap pole 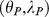, and the half angle that represents the size of the region in question 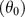. The SCHA model consists of a set of spherical cap harmonics, which can constitute a convenient orthogonal basis over a specific spherical cap 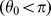, and it is expressed as follows:
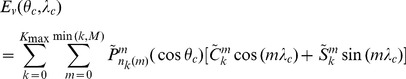
(1)where 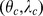 is the spherical cap coordinate of an Ionosphere Pierce Point (IPP), and is calculated using the geographical coordinate of the spherical cap pole and IPP;


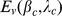
 is the vertical TEC at the IPP 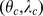;


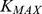
 and 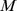 are the maximum degree and order of the series, respectively;


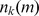
 are non-integer degrees of the orders (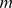), 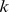 is the index of degrees;


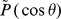
 is the normalized associated Legendre function;and 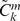 and 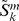 are normalized spherical cap harmonic coefficients.

The equation (1) has the similar formula expression of the global spherical harmonic function, while a major difference of them exists in the values of degrees [14]. In the case of spherical cap 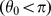, the degrees (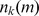) are non-integer, and they are a function of the orders (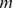) given a specific half angle 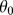, whereas the degrees of spherical harmonic function 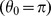 are simply natural numbers from 1 to 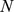. The calculation method of non-integer degrees given a half angle 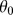 was given in the work [27].

The SCHA ionospheric model has been used by several research teams to map ionospheric TEC in different regions of the Earth, and it is suitable for large regions, particularly the polar areas, according to the comparisons between the SCHA method and the other regional models [27]–[34]. The zero-degree coefficient of the SCHA model represents the mean TEC of a specific region [14]–[16]. The mean TEC corresponds to an idealized ionosphere in which the TEC is uniformly distributed and, as a whole, has the same electron content as the actual ionosphere in the specific region; therefore, the mean TEC should represent the characteristics of the regional ionosphere [17]. The mean TEC has units of TECU



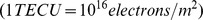
.

**Figure 1:**
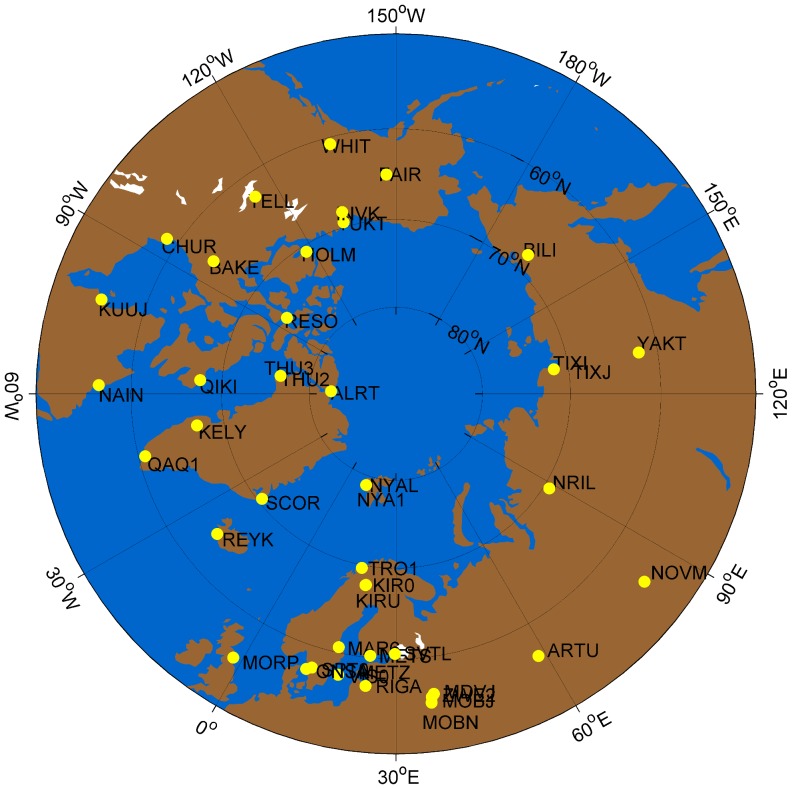
The geographical locations of the IGS tracking stations in the Arctic area. Land is indicated by brown, and sea/ocean is represented by blue. The yellow points indicate the locations of the IGS stations used in this study.

In the present study, the geographical North Pole is the spherical cap pole of the interested area, and the half angle is 30 degrees (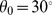), the maximum degree is 8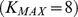 and the maximum order is 6 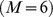. The number of model parameters is 75 in total. The Arctic ionospheric TEC is estimated using GPS measurements from 44 IGS tracking stations located at high latitudes (above 55° North latitude, as shown in Figure 1) and related IGS products, including IGS precise orbit data, and differential code bias (DCB) products of receivers and satellites provided by the Center for Orbit Determination in Europe (CODE) (ftp://ftp.unibe.ch/aiub/CODE). Table I listed the used IGS stations with their geographical coordinates. One should note that some IGS stations located in the Arctic were not included in Table I because their DCB products of receivers are missing from the database. Some pairs of stations have very close coordinates because the two receivers share the observation facility. The measurement dataset of the study period from 2000 to 2013 is provided in RINEX (Receiver Independent Exchange) format by the IGS central bureau via ftp access (ftp://cddis.gsfc.nasa.gov/gps/data/daily/). Before the study period, GPS tracking stations in the Arctic region are not sufficient to map ionospheric TEC. The sample rate of the GPS measurements is 30 seconds, and the elevation cut-off threshold is 20 degrees in the data processing. The sp3 satellite orbit products are used to calculate the precise positions of satellites and further calculate the positions of the ionosphere pierce points and elevations of GPS signal paths. The Spline interpolation method is used to interpolate the satellite positions at the observation epochs. Based on the estimated Arctic TEC, the analysis and prediction are presented as follows. The reference index data such as solar and geomagnetic indices have been downloaded from the national geophysical data center (ftp://ftp.ngdc.noaa.gov/STP/).

**Table 1:** The List and geographical locations of the IGS tracking stations in the Arctic area.

IGS name (4-char)	Latitude (degree)	Longitude (degree)	Elevation (meter)	IGS name (4-char)	Latitude (degree)	Longitude (degree)	Elevation (meter)
ALRT	297.6595	82.4943	78.11	MORP	358.3145	55.2128	144.40
ARTU	58.5605	56.4298	247.51	NAIN	298.3113	56.5370	33.48
BAKE	263.9977	64.3178	4.41	NRIL	88.3598	69.3618	47.89
BILI	166.4380	68.0761	456.24	NYA1	11.8653	78.9296	84.00
CHUR	265.9113	58.7591	−18.90	NYAL	11.8700	78.9300	82.00
FAIR	212.5008	64.9780	319.18	ONSA	11.9255	57.3953	45.50
HOLM	242.2391	70.7364	39.50	QAQ1	313.9522	60.7152	110.40
INVK	226.4730	68.3062	46.36	QIKI	295.9663	67.5593	13.30
KELY	309.0552	66.9874	229.81	RESO	265.1067	74.6908	34.90
WHIT	224.7779	60.7505	1427.00	REYK	338.0445	64.1388	93.10
VIS0	18.3673	57.6539	79.80	RIGA	24.0587	56.9486	34.70
YELL	245.5193	62.4809	181.00	SCOR	338.0497	70.4853	128.50
ZWE2	36.7601	55.7000	272.00	SPT0	12.8913	57.7150	219.90
KIR0	21.0602	67.8776	497.90	SVTL	29.7809	60.5329	77.10
KIRU	20.9684	67.8573	391.10	THU2	291.175	76.5370	36.10
KUUJ	282.2546	55.2784	−0.48	THU3	291.175	76.5370	36.10
MAR6	17.2585	60.5951	75.40	TIXI	128.8664	71.6345	46.98
MDVJ	37.2145	56.0215	257.40	TIXJ	128.8664	71.6345	47.05
METS	24.3953	60.2175	94.60	TRO1	18.9396	69.6627	138.00
METZ	24.3953	60.2175	94.50	TUKT	227.0057	69.4382	1.54
MOBJ	36.5697	55.1149	182.61	NOVM	82.9095	55.0305	149.98
MOBN	36.5695	55.1149	182.63	YAKT	129.6803	62.0310	103.37

## Analysis on Time-varying Periodograms of the Arctic TEC

This section first analyzes periodograms of the Arctic TEC variability over the past 14 years (4961 days) from 2000 to 2013. For purposes of comparison, the Global Ionosphere Maps (GIM) product of CODE is used to calculate the Arctic mean TEC for the same region and the same time period [35]. The same dataset of GIM has been utilized in the studies of [16]–[17], [36]. The GIM dataset represents global ionosphere TEC using a set of pre-defined grid points in the standard IONEX format [35]. As calculated by equation (2), GIM-derived regional mean TEC is the normalized weighted sum of TEC values of all IONEX grid points in the whole area of study.
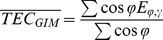
(2)where 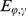 is the TEC grid value associated with the geographic latitude and longitude (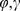) of the grid points, and 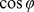 is the weighting function for grid points of the geographic latitude 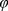. The sum of all weighting function 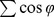 in the denominator is the normalization factor.

**Figure 2:**
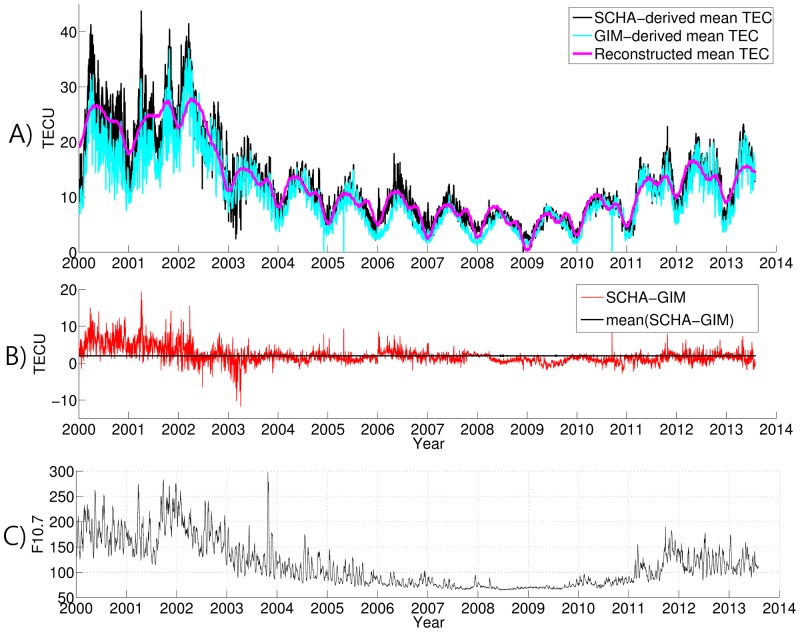
The Arctic TEC time series and corresponding solar activity condition of the time period. Top (A) Time series of the SCHA-derived Arctic mean TEC (black) and the GIM-derived Arctic mean TEC (cyan) and the reconstructed periodic oscillation component of the Arctic mean TEC based on the four periods (magenta). Middle (B) The difference between the SCHA- and GIM-derived mean TEC (cyan) and the mean of the difference (black). Bottom (C) The 10.7-cm radio flux for 2000–2013, indicating solar activity conditions.

The top panel of Figure 2 shows the time series of the SCHA-derived Arctic mean TEC (black line) and the GIM-derived Arctic mean TEC (cyan line). The two time series have a high correlation coefficient of 0.9613. Over the whole time period, the mean difference between the two time series is 2.01 TECU with a standard deviation of 2.20 TECU, as shown in the middle panel of Figure 2. The SCHA-derived Arctic mean TEC is larger than the GIM-derived result under active ionosphere conditions (2000–2003 and 2012–2013), which is indicated by the *F*10.7 index showed in the bottom panel of Figure 2, while the two time series are comparable under calm ionosphere conditions (2008–2009). This observation indicates that the GIM-derived Arctic mean TEC is “averaged” by the global coverage, which is consistent with the conclusions regarding the hemisphere and latitude-band distribution of the mean TEC in [16]–[17], [36].

In this study, the analysis and prediction are performed through the method of least-squares collocation, which is a generalization of least-squares adjustment, as presented in detail in [14]. The time series of the Arctic mean TEC over a given time interval is divided into the average TEC and a component of periodic oscillations with multiple periods, which is depicted mathematically using the harmonic expansion as follows [16]–[18]. 
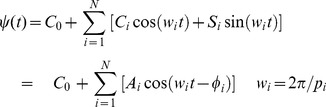
(3)where 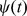 is the time series of the Arctic mean TEC for the sliding window, 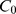 is the average TEC over a specific interval of the sliding window, 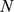 is the number of periods, 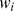 is the angular frequency with a period 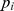, and 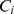 and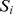 are parameters to be estimated, which define the phase and amplitude of the periodograms of the corresponding periodic oscillation components, 
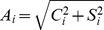
 is the amplitude of each periodic oscillation component, and 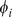 is the corresponding phase.

**Figure 3:**
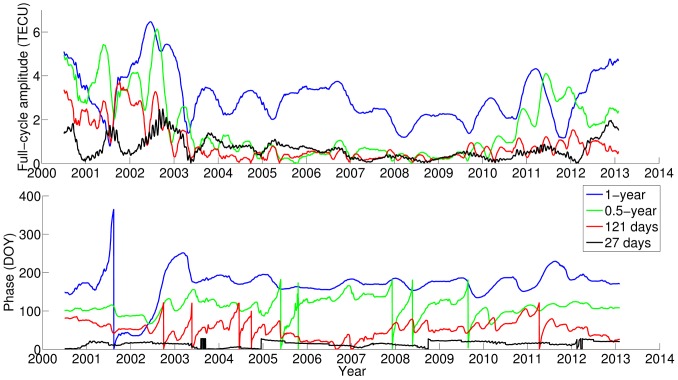
The time-varying periodograms of the selected periodicities of the Arctic TEC. Time evolution of the periodograms, including the full-cycle amplitude (top) and phase (bottom), for the four selected periods of the Arctic mean TEC.

In this study, a sliding window of one year is used, and the four selected periods 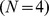 include annual, semiannual, terannual and 27-day cycles. The sine and cosine coefficients (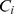 and 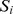) in equation (3) allow for a determination of both the phase and amplitude of each periodic oscillation component. As shown in Figure 3, the significant periodic variability in the phase and amplitude of the periodograms allows the oscillation components of the Arctic mean TEC to be predicted by simply extrapolating the periodograms.

In this study, the frequency spectra of the time-varying periodograms of the four oscillation components are analyzed separately, and the three most significant periods for the periodograms of each oscillation component are estimated. The three most significant periodic components represent more than 92% of the power in the frequency spectrum. Table II presents the significant periods and normalized powers of the corresponding periodograms. The spectral peaks near 27 days are due to sunspots co-rotating with the Sun's surface; these peaks spread over a certain range of periods because the angular velocity of sunspot rotation varies with solar latitude. In this study, we use a period of 27.8 days, which is the mean of the range associated with the spectral peaks. Compared to the SCHA-derived mean TEC, the time series of the TEC reconstructed using the time-varying periodograms has a Root Mean Square (RMS) error of 3 TECU under active ionosphere conditions and 1.2 TECU under calm ionosphere conditions, which correspond to approximately 10% of the ionosphere TEC, as shown in Figure. 2A).

**Table 2:** Three Significant Periods and Normalized Powers of The Periodograms of The Oscillation Components of the Arctic Mean TEC.

Oscillation components	Period and power (in parentheses) of the periodograms		
Annual oscillation	1 year (0.622)	2 years (0.243)	11.22 years (0.135)
Semiannual oscillation	0.5 years (0.789)	1 year (0.059)	11.22 years (0.152)
Terannual oscillation	121.7 days (0.726)	1 year (0.046)	11.22 years (0.228)
27-day oscillation	27.7 days (0.806)	1 year (0.016)	11.22 years (0.178)

**Figure 4:**
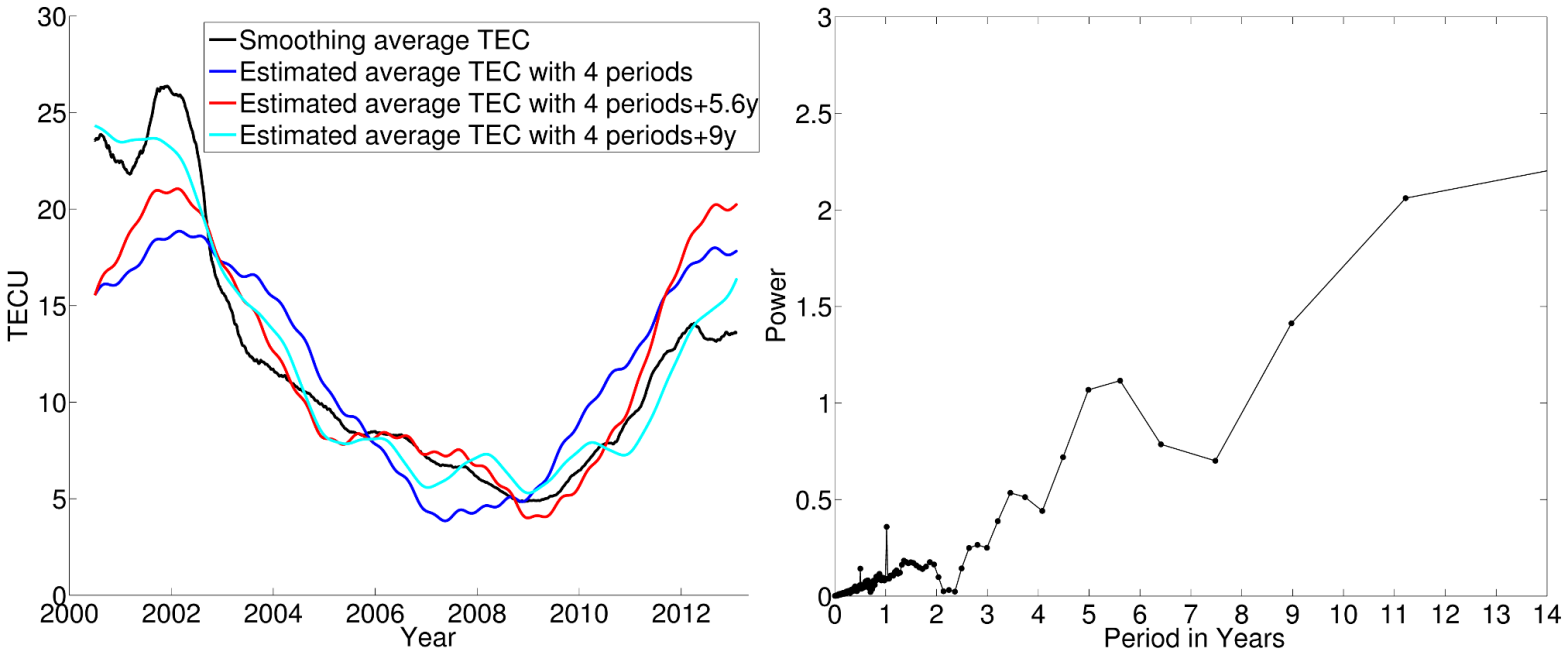
The time series and spectrum of average TEC related to different selections of periods. Left: Time series of the smoothed average TEC based on the sliding window (black), the average TEC reconstructed using the four well-established periods (blue) and with the inclusion of the periods of 5.6 years (red) and 9 years (cyan). Right: Periodic spectrum of the time series of the average TEC estimated with the sliding window.

In addition to the oscillation components, the average TEC estimated from the sliding window shows the variability over long-term periods. Figure 4 shows the time series of the average TEC (labeled as “Smoothing average TEC”) and its periodic spectrum. In addition to the well-established spectral components, with periods of 11.22 years, 2 years, 1 year and 0.5 years, the spectrum also includes unexpected periods, e.g., 5.6 years and 9 years [16]. Figure 4 shows the time series of the average TEC reconstructed using the well-established four periods alone and in combination with the two unexpected periods. Because the four periods of 11.22 years, 2 years, 1 year and 0.5 years are established according to general ionosphere physics and the average Arctic TEC of one-year-length sliding window has spectral components other than these periods, the reconstructed TEC does not fit exactly with the original TEC time series [16].

Some of these major periods are in accordance with well-established physical processes. The 11.22 years period is related to the cycle of solar sunspots. Both components of the annual and semi-annual periods have long been understood [16], [37]. The 5.6 years period could be interpreted as a second Fourier harmonic of the 11.22 years solar activity cycle, which is related to the asymmetry of the TEC time series. The 9 years period could be an artifact of the recent unusually long solar activity minimum (solar cycle 23–24 minimum). The following section examines the results of backward prediction involving the 5.6 years and 9 years periods, respectively.

## Results of Long-term Prediction of the Arctic TEC

Prediction of the Arctic mean TEC is accomplished by summing the predicted oscillation components and average TEC, which are calculated separately based on their respective periodograms. This section presents backward and forward predictions of the Arctic mean TEC over the scale of a solar cycle. The backward prediction provides an opportunity to verify the reliability of the prediction method by comparing the predicted results with past observations, although the prediction process does not require any physical observations over the time interval of prediction.

**Figure 5:**
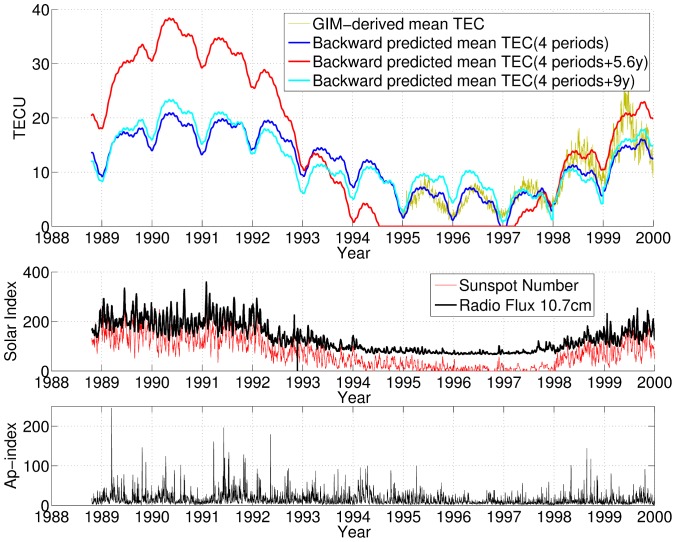
Backward predicted Arctic mean TEC for 1988–1999 and the corresponding geographical indices. Top: Backward predicted Arctic mean TEC for 1988–1999 based on different period sets of the average TEC, in addition with the GIM-derived Arctic mean TEC for 1995–1999. Negative TEC values are set to zero. Middle: Solar indices for 1988–1999, including the sunspot number and the 10.7-cm radio flux. Bottom: The geomagnetic index (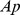) for 1988–1999.

The four oscillation components are extrapolated temporally based on their time-varying periodograms, as discussed in Section 3. For the average TEC prediction, this study compares the results of three sets of selected periods: *A*) the four well-established periods: 11.2 years, 2 years, 1 year and 0.5 years; *B*) the above four periods plus the 5.6 years period; *C*) the above four periods plus the 9 years period. Figure 5 shows the results of the backward prediction, which are the summation of the predicted oscillation components and the average TEC, for the preceding 11.2 years, from October 1988 to December 1999. The prediction results are set to zero when the values are negative. For purposes of comparison, Figure 5 also displays indices that are strongly correlated to the ionosphere for the same time period, including the solar sunspot number, the 10.7-cm solar radio flux and the geomagnetic index 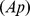, which are used to examine the results of the backward prediction of the Arctic mean TEC.

The coefficients of correlation between the predicted mean TEC and the geophysical indices provide a measure of evaluation; as a baseline, we use the correlation coefficients between the SCHA-derived Arctic mean TEC and the geophysical indices for 2000–2013. The baseline correlation coefficients are 0.8427 for *F*10.7 and 0.8205 for the sunspot number. The reconstructed mean TEC for 2000–2013 based on period set *C* has the largest correlation coefficients, 0.8117 for *F*10.7 and 0.8057 for the sunspot number, while the mean TECs reconstructed using period sets *A* and *B* have lower correlation coefficients of approximately 0.7 for the both solar indices. For the predicted Arctic mean TEC for 1988–1999, the results obtained for period sets *A*, *B* and *C* have comparable correlation coefficients of 0.7–0.75 for both solar indices over the same time interval. It should be noted that the prediction result of period set *B* has negative TEC values in the time 1994 to 1997, which is meaningless in physics and hence has been set to zero in Figure 5, although it is reasonable in mathematics that a prediction comes to a negative value due to prediction uncertainty when its true value is close to zero [38].

The Arctic ionospheric TEC has a low correlation coefficient of 0.1602 with the geomagnetic index (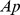). This result is consistent with the conclusions related to hemispherical and global scales, which are that the geomagnetic index is only related to the ionosphere over short-term periods, exhibiting a low correlation coefficient of approximately 0.2 with the long-term mean ionosphere [16]–[17].

This study calculates the GIM-derived Arctic mean TEC for 1995–1999 for comparison and accuracy evaluation. No data prior to this period are available. Table III compares the correlation coefficients for different time series of the SCHA-based and GIM-derived Arctic mean TEC. The results of backward prediction based on period sets *A* and *C* have correlation coefficients of more than 0.85 for the time series of the GIM-derived Arctic mean TEC, but the results for period set *B* have a significantly lower correlation coefficient of 0.4582. Compared to the GIM-derived Arctic mean TEC, the backward prediction results based on period sets *A* and *C* have RMS errors of 3.6 TECU and 3.1 TECU, respectively, for the entire duration of five years. The prediction results for period set *B* include negative TEC values for 1994 to 1997. For the entire duration of 11.2 years, the prediction accuracy for period sets *A* and *C* is estimated using the covariance of the parameters in the least-squares collocation [14], [25]. The prediction uncertainty, which is depicted by the standard error of predicted values, is found to be 5.6 TECU for period set *A* and 4.8 TECU for period set *C*, which correspond to 25% of the average TEC under active ionosphere conditions. The discrepancy in the prediction results for the two period sets is within the prediction uncertainties.

**Table 3:** Correlation Coefficients for the SCHA-based TEC Time Series and the GIM-derived Arctic Mean TEC.

TEC time series	GIM-derived Arctic mean TEC	
	2000–2013	1995–1999
SCHA-derived daily mean TEC	0.9613	—
Reconstructed Arctic mean TEC for period set A	0.9044	0.8768
Reconstructed Arctic mean TEC for period set B	0.9125	0.4582
Reconstructed Arctic mean TEC for period set C	0.9183	0.8890

The prediction results obtained for period set *C*, which includes the 9 years period, exhibited the maximum correlation with the observed solar indices and the GIM-derived mean TEC for 1995–1999; it also displayed the minimum RMS error with respect to the GIM-derived mean TEC for 1995–1999. The 9 years period may arise from the exceptionally prolonged solar cycle 23, lasting from 1996 to 2008 [39]. In fact, historical data of sunspot numbers since 1740 have shown that the length of a solar cycle varies from 9 years to 14 years [40]. It need be validated whether the 9 years period exists commonly in the Arctic TEC using a longer dataset, and the driving force of this variability should be physically interpreted.

**Figure 6:**
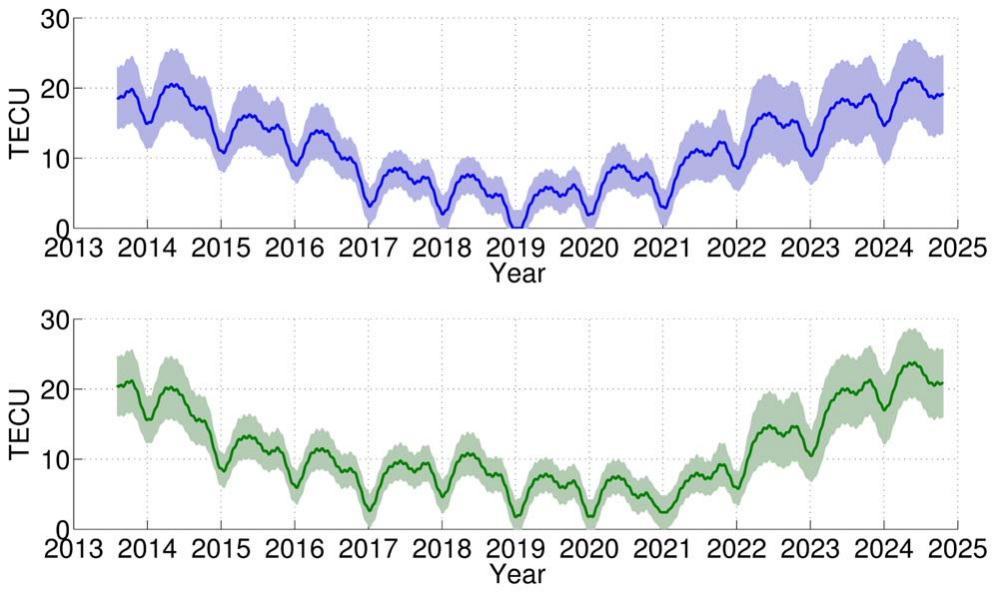
Forward predicted Arctic mean TEC and corresponding uncertainties for 2014–2024 using two period sets. Forward prediction and the corresponding uncertainty, indicated by the shaded band, based on different period sets of the average TEC: period set *A* (top) and period set *C* (bottom).

Based on the two period sets *A* and *C*, the Arctic mean TEC is predicted for the following 11.2 years, from August 2013 to 2024, as shown in Figure 6. Both predictions display significant variability, such as the 11.2 years, annual, semi-annual and seasonal variations. The prediction uncertainty is the same as that of the backward prediction, and the prediction confidence is indicated by the shaded band, which becomes wider towards the next 11.2 years maximum. The both time series of predicted TEC based on period sets *A* and *C* show that the Arctic TEC will reach its next minimum in December 2018 and its next maximum in the summer of 2024, after the previous ionosphere maximum occurring in May 2013.

## Conclusions

The dataset of the continuously operating GPS tracking stations in the Arctic region provides an unprecedented observation source of the Arctic ionospheric TEC in large spatiotemporal scales. In this study, a new method has been developed for predicting the Arctic TEC in the scale of a solar cycle using the past GPS dataset. The proposed method represents the time series of Arctic TEC with a component of periodic oscillations and a component of the average TEC. The periodograms of the variability of the Arctic TEC are then analyzed using a dataset for the past 13.6 years, from 2000 to 2013. The periodograms displayed time-varying evolution. Based on the time-varying periodograms, the newly developed method predicts the Arctic TEC over the scale of a solar cycle (11.2 years) using the technique of least-squares collocation. The prediction is performed in both temporally backward and forward directions. The backward predictions for the preceding solar cycle from 1988 to 1999 are compared to evaluate the performance with past physical data, including solar indices, the geomagnetic index and the GIM-derived Arctic TEC.

The proposed method of TEC prediction is based on the extrapolation approach that requires no input of physical observations of the time interval of prediction, and it is performed by summing the predicted periodic oscillation and average TEC components. The challenge in conducting long-term predictions of the ionosphere primarily arises in predicting the average TEC component. The proposed prediction method is verified using the backward prediction results, which show that TEC prediction result of involving the 9 years period is more consistent with the historical Arctic TEC dataset and geophysical data for the study duration. However, the 9 years period requires further confirmation and physical interpretation using a longer dataset because the duration of 14 years is a relatively short time, which represents all of the data available currently. Forward prediction for the future solar cycle from 2013 to 2024 is performed for two period sets of the average TEC. The prediction results for the two sets are consistent overall, with a standard error of 5.6 TECU and 4.8 TECU, respectively, which is equal to roughly 25% of the Arctic mean TEC under active ionosphere conditions.
